# NFkB disrupts tissue polarity in 3D by preventing integration of microenvironmental signals

**DOI:** 10.18632/oncotarget.1451

**Published:** 2013-10-14

**Authors:** Sabine Becker-Weimann, Gaofeng Xiong, Saori Furuta, Ju Han, Irene Kuhn, Uri-David Akavia, Dana Pe'er, Mina J Bissell, Ren Xu

**Affiliations:** ^1^ Life Sciences Division, Lawrence Berkeley National Laboratory1, Berkeley, CA; ^2^ Markey Cancer Center, University of Kentucky, Lexington; ^3^ Department of Molecular and Biomedical Pharmacology, University of Kentucky, Lexington; ^4^ Department of Biological Sciences, Columbia University, New York, NY

**Keywords:** Disorganization gene signature, p65, RelB, three-dimensional tissue structure, tissue polarity

## Abstract

The microenvironment of cells controls their phenotype, and thereby the architecture of the emerging multicellular structure or tissue. We have reported more than a dozen microenvironmental factors whose signaling must be integrated in order to effect an organized, functional tissue morphology. However, the factors that prevent integration of signaling pathways that merge form and function are still largely unknown. We have identified nuclear factor kappa B (NFkB) as a transcriptional regulator that disrupts important microenvironmental cues necessary for tissue organization. We compared the gene expression of organized and disorganized epithelial cells of the HMT-3522 breast cancer progression series: the non-malignant S1 cells that form polarized spheres (‘acini’), the malignant T4-2 cells that form large tumor-like clusters, and the ‘phenotypically reverted’ T4-2 cells that polarize as a result of correction of the microenvironmental signaling. We identified 180 genes that display an increased expression in disorganized compared to polarized structures. Network, GSEA and transcription factor binding site analyses suggested that NFkB is a common activator for the 180 genes. NFkB was found to be activated in disorganized breast cancer cells, and inhibition of microenvironmental signaling via EGFR, beta1 integrin, MMPs, or their downstream signals suppressed its activation. The postulated role of NFkB was experimentally verified: Blocking the NFkB pathway with a specific chemical inhibitor or shRNA induced polarization and inhibited invasion of breast cancer cells in 3D cultures. These results may explain why NFkB holds promise as a target for therapeutic intervention: Its inhibition can reverse the oncogenic signaling involved in breast cancer progression and integrate the essential microenvironmental control of tissue architecture.

## INTRODUCTION

Cancer is an organ specific disease. It is widely accepted that the loss of growth regulation is the central mechanism shared by all cancers. We have argued that the tissue architecture is at least an equally important regulator of malignant transformation, and one that may explain organ specificity of cancer [1, 2]. Disruption of polarized tissue structure [3-5] and induction of cellular invasion are accompanied by extensive changes in the cellular microenvironment; in many cases the basement membrane is degraded, cell-extracellular matrix (ECM) adhesion is altered and growth-regulatory signals are lost as a result. This microenvironmental remodeling and the consequent changes in tissue structure contribute to the development of a malignant phenotype [5, 6]. Over two decades ago we developed a 3-dimensional (3D) culture system using laminin-rich ECM (lr-ECM) to distinguish normal and malignant breast phenotypes [7]. In 3D cultures, non-malignant HMT-3522 S1 (S1) cells form polarized, growth-arrested spheres, which closely resemble the alveolar structures of healthy breast tissues referred to as acini [8]. We have extended the series to characterize premalignant phenotype of additional cell-lines referred to as S3 series [9]. We have used these assays to identify and characterize the microenvironmental factors and signals controlling acquisition of a malignant phenotype and its reversal [7, 10-12] (for further references see [2]). The isogenic malignant counterpart of S1 cells, the HMT-3522 T4-2 (T4-2) cells, display an altered profile of microenvironmental receptors and downstream signaling, and form disorganized, proliferative structures displaying enhanced cell invasion under the same conditions [10, 12] (and references cited above).

In addition to differences in tissue organization, compared to their S1 counterparts, the malignant T4-2 cells have significantly higher expression of epidermal growth factor receptor (EGFR), phosphatidylinositol 3-kinase (PI3K), mitogen-activated protein kinase (MAPK) as well as beta1-integrin and matrix metalloproteinases (MMPs) such as MMP9 among others [5, 9, 10, 12]. We have repeatedly shown that by manipulating the microenvironmental signals in T4-2 and other malignant cells [12], the cells can be induced to form polarized, non-invasive structures resembling those formed by the non-malignant S1 cells (‘T4-2R’ cells). We have referred to this phenomenon as ‘phenotypic reversion’ [10].

Decreasing activation of any of the above pathways down-modulates their downstream signals, normalizes the signaling through all the other pathways and reverses the malignant phenotype of T4-2 cells in 3D culture. We know also that changes in glucose uptake play an equally important role in both disorganization and reversion [13]. Thus many seemingly disparate agents and mechanisms induce a common phenotypic change (reversion of the chaotic malignant phenotype to an organized acinar-like structure) if the cells are in a physiologically relevant 3D microenvironment.

This observation led us to hypothesize that depending on the microenvironmental signaling, the cells must experience changes in their transcriptional programs that accompany the observed changes in colony architecture. We also hypothesized that there would be one or more common transcriptional regulators that integrate the signals to effect this switch. To find such regulators, we used microarray data of non-malignant S1 and malignant T4-2 cells, as well as data that had been obtained for T4-2 cells ‘phenotypically reverted’ by the widest variety of inhibitors, and compared the common denominators. These analyses identified the transcription factor nuclear factor-kappa B (NFkB) as a prominent switch for regulating the change between disorganization and polarity in response to microenvironmental changes.

NFkB regulates a number of cancer-related processes including immune-response, inflammation, cell survival and cancer progression [14]. The NFkB family consists of five structurally and functionally conserved members in mammalian cells, including RELA (p65), RELB (p60), c-REL, NFkB1 (p105 and p50), and NFkB2 (p100 and p52) [15]. Elevated NFkB binding activity has been observed in both primary human breast cancer tissues and breast cancer cell lines, and contributes to the activation of CYCLIN D1, c-MYC, and MUC1 [16-18]. Despite considerable literature suggesting the importance of NFkB in cancer in general and in breast cancer in particular, the reasons behind its dominant activation in tumors are still undefined. We find that NFkB acts as a transcriptional rheostat the level of which directs a phenotype of order and its aberrant expression spells architectural disorder in response to microenvironmental changes. This finding could provide a fine-tuning of a framework for designing more compelling therapeutics.

## RESULTS

### Comparison of malignant disorganized colonies with organized acini identified a set of genes common in reversion

**Figure 1:**
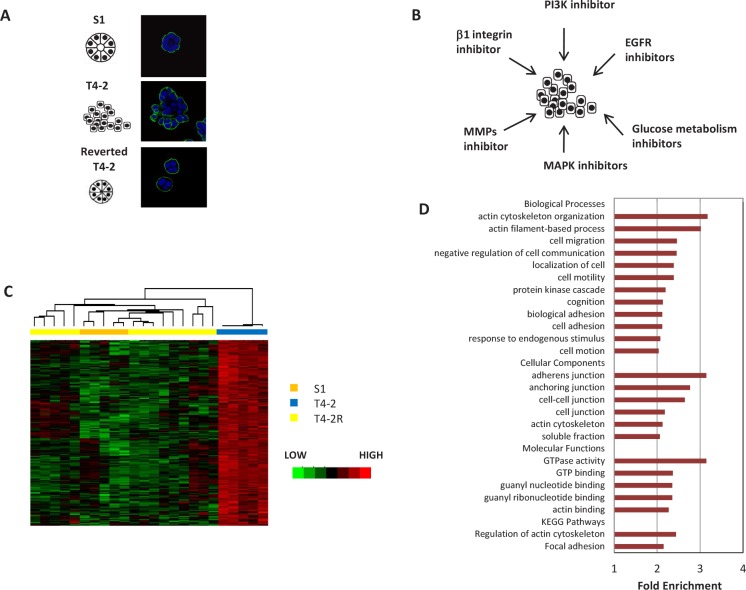
A cluster of genes revealed by microarray analysis is associated with tissue polarity in HMT3522 cell lines A) Immunofluorescence analysis of basal marker alpha6-integrin in S1, T4-2, and T4-2 cells phenotypically reverted in 3D culture. S1 and reverted T4-2 cells formed polarized spheroids, but T4-2 cell formed disorganized structures. Blue:DAPI, green: alpha6 integrin. Scale: 25μm B) Scheme showing the pathways that were blocked to reverse the malignant phenotypes of T4-2 cells in 3D culture for the microarray analysis. C) Unsupervised clustering of the 180 genes upregulated in T4-2 cells as compared to S1 cells and reverted T4-2 cells. D) Gene Ontology terms (biological processes, cell components, molecular functions) and KEGG pathways overrepresented in the disorganization gene signature.

We showed previously that the modification of aberrant microenvironmental signaling in malignant human mammary epithelial cells (HMECs) reprograms them to form polarized, growth-arrested structures in 3D cultures [10, 12] (Figure 1A). To identify the alterations in gene expression profiles associated with such reprogramming events, we utilized microarrays of S1, T4-2, and T4-2R cells reverted by inhibitors of EGFR, beta1-integrin, MAPK, PI3K and by broadband inhibitors of MMPs as well as by reduced glucose metabolism (Figure 1B and Supplemental Table 1, GEO database, GSE50444). We obtained a gene expression matrix with data from 6560 genes and 24 microarrays – 5 of S1, 5 of T4-2 and 14 of T4-2, reverted with different agents (Supplemental Table 2). Unsupervised hierarchical clustering analysis showed that S1, T4-2 and T4-2R cells each cluster together (Supplemental Figure S1).

We identified a set of 345 genes that were differentially expressed in cells with different 3D phenotypes – polarized or disorganized – (LIMMA/R Bioconductor, p=0.005). The genes upregulated in T4-2 cells (180 genes) were called ‘disorganization gene signature’ (Supplemental Table 3). Genes that were upregulated in S1 cells and reverted T4-2 cells (165 genes) were referred to as ‘organization gene signature’. We used the disorganization gene signature for further analysis in order to find transcriptional regulators involved in the disturbance of acinar structure in T4-2 cells. The expression and hierarchical clustering of these genes is depicted in Fig. 1C.

Using the statistics gene ontology tool DAVID [19], we identified genes encoding proteins located in adherens junctions and the actin cytoskeleton as well as genes involved in processes such as cell adhesion, focal adhesion and actin cytoskeleton organization, that were significantly enriched in the disorganization gene signature (Figure 1D). We also found that genes involved in cell migration were overrepresented, consistent with a previous report correlating disruption of tissue polarity with endothelial cell migration [20]. Furthermore, gene set enrichment analysis (GSEA, [21]) of the gene expression matrix confirmed an increase in expression of actin organization in T4-2 cells (p=0.002).

### NFkB associated with the expression of disorganization genes

**Figure 2:**
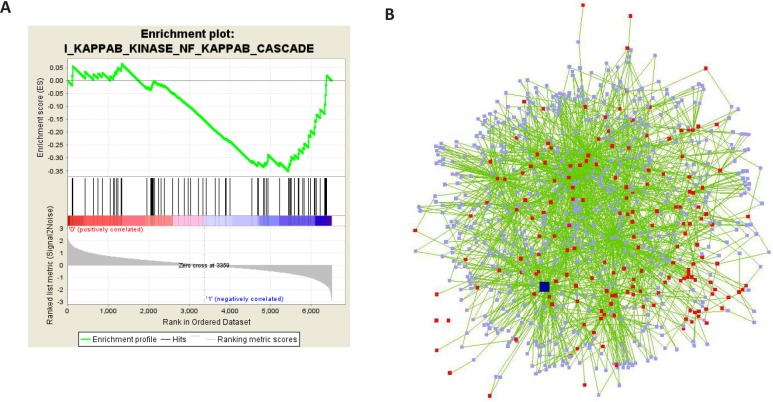
GSEA and network analyses reveal NFkB as a potential regulator of disorganization genes A) Gene set enrichment analysis [21] finds the gene set ‘positive regulation of IKK and NFkB cascade’ to be enriched among genes that show a high expression in unorganized cells (1:'negatively correlated') and low expression in organized cells in the microarrays (0:'positively correlated') (p=0.09). B) A regulatory network consisting of the 180 genes of the disorganization signature as well as connecting nodes that interact with at least two disorganization genes was identified using Cytoscape. Red nodes: disorganization genes, light blue nodes: connecting genes, green edges: interactions found in NCBI, dark blue node: p65.

In order to identify common signaling pathways and/or transcription factors involved in the disorganization of the 3D structures, we employed several bioinformatics tools. Comparison of HMT-3522 expression profiles with identified gene sets (Gene Set Enrichment Analysis, [21]) revealed that genes related to the ‘positive regulation of IKK and NF-κB cascade’ are activated in disorganized T4-2 cells (p<0.09, Figure 2A). Using the network analysis tool Cytoscape, we identified a regulatory network associated with the differentially expressed genes. p65, i.e. one of the subunits of NFkB, was one of the major hubs of the network with more than 100 neighbors (Figure 2B). Most notably, transcription factor binding site analysis of the disorganization cluster using TransFind (transfind.sys-bio.net/, [22]) identified NFkB as a potential transcription factor for the disorganization genes with the third highest significance (p<0.005); 24 of the 180 genes were identified as potential NFkappaB target genes (Supplemental Table 3, marked in yellow). This corresponds to a percentage 1.8 times as high as in the total set of 6560 genes.

These results suggest that NFkB is a potential regulator of the disorganization genes, and that activation of NFkB may be associated with disruption of polarized mammary tissue structure.

### Expression and activation of NFkB is associated with the disorganized tissue phenotype

**Figure 3:**
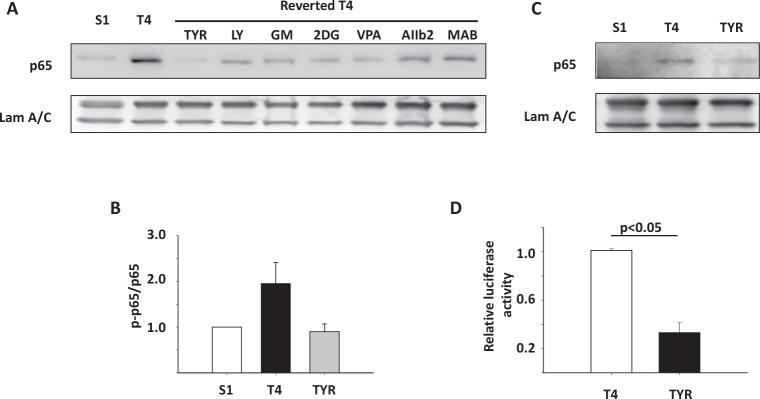
The NFkB pathway is activated in disorganized T4-2 cells compared to polarized S1 and reverted T4 cells A) Immunoblotting analysis of p65 expression in S1, T4-2, and reverted T4-2 cells in 3D culture. Laminin A/C was used as a loading control. T4-2 cells were reverted by tyrphostin (Tyr), an EGFR inhibitor; LY294002 (LY), a PI3K inhibitor; GM6001 (GM), a MMP inhibitor; 2DG, a glucose metabolism inhibitor; VPA, a HDAC inhibitor; AIIB2, a β1 integrin blocking antibody, MAB225 (MAB), an EGFR blocking antibody. B) Immunoblotting analysis of phosphorylated p65 in S1, T4-2, and T4-2 cells reverted by tyrphostin. The blot results were quantified by AlphaEaseFC software, and expressed as relative levels of phosphorylated p65 to total p65. C) Immunoblotting of nuclear protein extracted from S1, T4-2, and T4-2 cells reverted with tyrphostin. D) Luciferase analysis of NFkB transcription activities in T4-2 and reverted T4-2 cells. Graph displays average ± SEM; n=4.

To confirm that NFkB is indeed activated in disorganized MECs compared to polarized cellular structures, we assessed the expression of the NFkB subunit p65 in S1, T4-2, and reverted T4-2 cells. We found that mRNA levels of *p65* were significantly upregulated in disorganized T4-2 cells compared to polarized S1 cells or reverted T4-2 cells. Interestingly, *p65* is one of the 180 genes of the disorganization signature in T4-2 cells (Supplemental Figure S2). Among the 25 genes with the highest correlation of expression with *p65*, there were three genes involved in ECM-receptor interaction, alpha6-integrin, beta4-integrin and thrombospondin (with correlation coefficients of 0.91, 0.87 and 0.86 respectively), indicating a tight connection between their expression and *p65* (data not shown). *p65* was also increased at the protein level in untreated T4-2 cells compared to S1 cells. Reversion of T4-2 cells by blocking of EGFR, beta1-integrin, MMP, and their downstream signals drastically reduced the expression of *p65* in those cells (Figure 3A), suggesting that microenvironmental cues contribute to NFkB activation in the disorganized HMECs.

It has been reported that p65 is phosphorylated at Ser536 by IkappaB kinase (IKK), NFkB activating kinase, and Ribosomal S6 kinase 1 (RSK1), which in turn induces nuclear translocation of p65 [23]. Thus, we analyzed the phosphorylation at Ser536 and nuclear levels of p65. Quantification of immunoblotting results showed that Ser536 phosphorylation of p65 was increased in T4-2 cells and repressed in S1 and reverted T4-2 cells (Figure 3B). In addition, nuclear translocation of p65 was consistent with phosphorylation status (Figure 3C). In order to assess the NFkB activity in T4-2 and reverted T4-2 cells directly, we measured the luciferase activity in cells stably transfected with a luciferase reporter construct that is controlled by NFkB response elements. We found that the transactivation activities of NFkB were significantly reduced in T4-2 cells reverted by the EGFR inhibitor (Figure 3D). In summary, we find the malignant phenotype associated with higher levels of *p65* mRNA, p65 protein levels as well as increased NFkappaB transcriptional activity.

### Downmodulation of NFkB leads to phenotypic reversion

**Figure 4:**
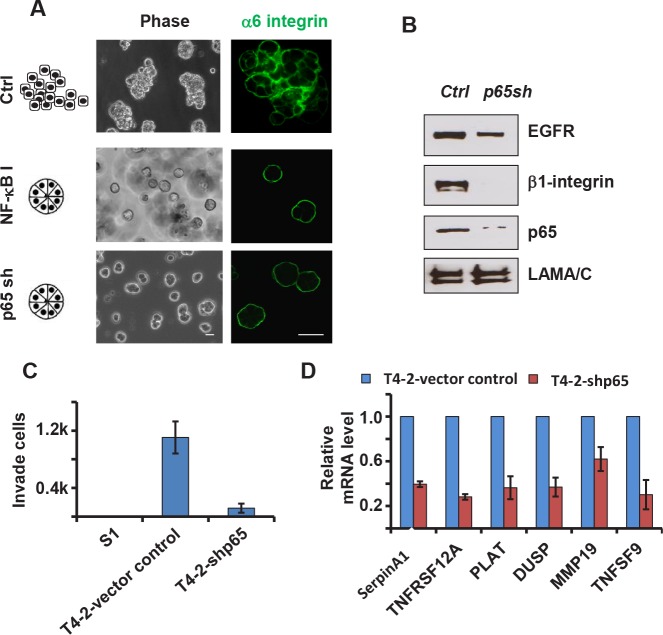
Inhibition of the NFkB pathway reverses the malignant phenotypes of T4-2 cells in 3D culture A) Phase and immunofluorescence images of T4-2 cells. Cells were either treated with NFkB inhibitor (Wedelolactone, 10 μM) and cultured in 3D for four days orinfected with p65 shRNA lentivirus before plating in 3D culture. Blocking the NFkB pathway reprograms the cells to form polarized spheroids structure. Green: alpha6-integrin. Scale: 25μm. B) Immunoblotting analysis of beta1-integrin, EGFR and p65 expression in control and p65 shRNA-expressing T4-2 cells. C) Transwell invasion analysis of control and p65 shRNA-expressing T4-2 cells. Graph displays average ± SEM; n=3. D) Quantification of mRNA levels of NFkB target genes by real-time PCR. Control and p65 shRNA-expressing T4-2 cells were cultured in 3D for 4 days before RNA extraction.

To show that inhibition of NFkB indeed leads to phenotypic reversion, we blocked NFkB activation in T4-2 cells by two means. T4-2 cells cultured on top of 3D laminin-rich ECM (lrECM) gels for four days were treated with Wedelolactone, an IKK inhibitor [24]. The Wedelolactone-treated cells formed small spheroid structures with basal polarity (alpha6-integrin), whereas the vehicle-treated control cells remained as disorganized, larger structures (Figure 4A). We also utilized shRNA to reduce p65 expression in T4-2 cells (Figure 4B). Reducing p65 expression reprogrammed T4-2 cells to form polarized spheroids structures in 3D cultures (Figure 4A), and cell invasion was significantly suppressed (Figure 4C). Using quantitative RT-PCR, we measured several NFkB -targeted genes to compare the NFkB activity in control vs. *p65* knockdown cells. Our results showed significant reduction in mRNA levels of *SERPINA1, TNFSF12A, PLAT, DUSP, MMP19*, and *TNFSF9* after downregulation of *p65* (Figure 4D). As expected from our previous data, we found that the protein levels of beta1-integrin and EGFR were also decreased in *p65* knockdown cells (Figure 4B). NFkB provides a ‘disintegration node’ in a 3D tissue-like setting.

### The expression of the disorganization genes correlates also with the phenotype of other breast cancer cell lines in 3D

**Figure 5:**
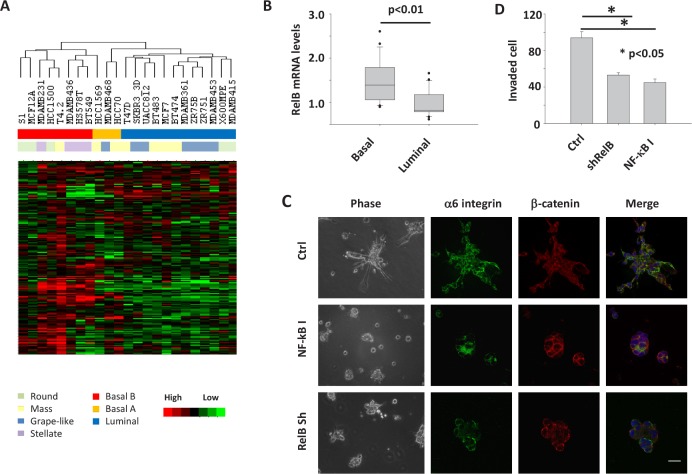
Expression of disorganization genes and activation of the NF-κB pathway are associated with aggressive 3D phenotypes of multiple breast cancer cell lines A) Unsupervised hierarchical clustering of breast cancer cell lines in 3D culture using the 180 disorganization genes. Each row represents a gene; each column represents a cell line. B) Bar graph of RelB mRNA levels in basal and luminal types of breast cancer cell lines. RelB mRNA expression is assessed by Affymetrix microarray and measured as log2 (probe intensities). C) Phase and immunofluorescence images of BT549 cells. Cells were either treated with Wedelolactone in 3D culture for 4 days or infected with RelB shRNA retrovirus before being seeded in 3D culture. D) Transwell invasion analysis of BT549 cells. The cells were treated with NF-κB inhibitor (Wedelolactone, 10 μM) or transfected with RelB shRNA vector.

The 3D morphologies of a substantial panel of human breast cancer cell lines correlate with their gene expression profiles in 3D cultures and tumor cell invasiveness [25]. Since mRNA levels of the 180 disorganization genes are associated with tissue structure in the HMT-3522 cell lines, we sought to determine whether these genes are similarly associated with 3D morphologies of other breast cancer cell lines. Comparing the average expression of the disorganization genes in 24 breast cancer cells lines, we found a higher expression in basal cell lines than in luminal cell lines (Supplemental figure S3). Unsupervised hierarchical clustering analysis of the disorganization gene set revealed a correlation of expression with subtype as well as 3D morphology. Basal B cell lines clustered together with higher expression of disorganization genes compared to luminal cell lines. Within the basal B cluster (I), the majority of the cancer cells with stellate morphology grouped together (Figure 5A). Most of the cell lines in the second cluster (II) are luminal and basal A subtypes with spheroid 3D, mass or grape-like phenotypes. The majority of basal B cells form stellate structure in 3D culture and lack E-cadherin expression, reflecting decreased cell–cell interactions and enhanced invasion [25]. Therefore, the up-regulation of disorganization genes seems associated with aggressive morphologies and increased cell invasion in 3D cultures.

To examine whether NFkB activity is functionally involved in the 3D phenotypes of other breast cancer cell lines, we chose BT549, a basal cancer cell line with a stellate morphology. It was treated with Wedelolactone in 3D culture. Most of the Wedelolactone-treated cells formed round or mass structures in 3D lrECM gels (Figure 5C). The colony structures were more organized in drug-treated cells as shown by beta-catenin and nuclear staining, although the metastatic cells were not fully polarized (Figure 5C), as was the case for MDA-MB-231 cells which require more than one inhibitory factor to be fully reverted [12]. The dramatic changes in tissue structures were accompanied by a significant reduction in cell invasion (Figure 5D). Since mRNA levels of the NFkB subunit *RELB* are elevated in basal-type breast cancer cells (Figure 5B), NFkB signals in those cells can also be blocked with shRNA targeting the *RELB* gene. We found that in 3D culture, *RELB*-silenced BT549 cells form structures with reduced invasive branches which we termed ‘mass’ (Figure 5C and supplemental Figure S4) [25]. In addition, knockdown of *RELB* significantly inhibited invasion of BT549 cells (Figure 5D). These results indicate the involvement of NFkB pathway in invasion of basal-type breast cancer cells in 3D gels.

**Figure 6:**
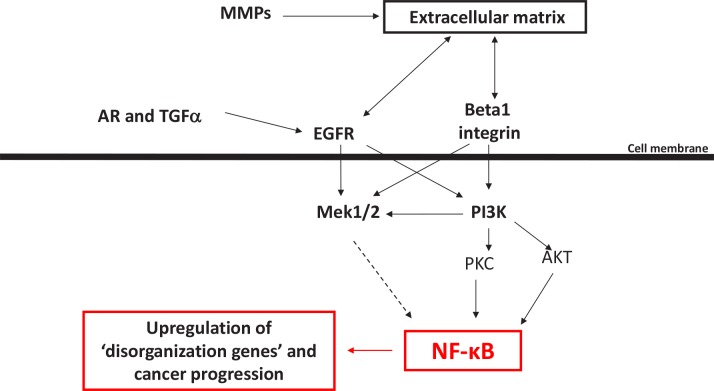
A scheme showing the role of NF-κB as potential integrator of various microenvironmental cues and regulator of multicellular organization

## DISCUSSION

Loss of tissue organization is a trait of every malignant tumor, but growth rather than architecture has traditionally been accepted to be the most important hallmark of cancer. Yet for more than a decade we have shown repeatedly that the architecture of the breast acini in 3D cultures can be dominant over the malignant genome, i.e. the cancer cell phenotype and growth status can be reversibly altered if the architecture is restored via microenvironmental signaling [1, 2]. This reversibility in the absence of genetic changes [26] implies that the phenotype is linked to the regulatory status and relatively unaffected by the genetic composition of the cells. Interestingly, within the same malignant cells, the inhibition of many different signaling pathways leads to a similar organized phenotype [3]. Because of reversibility and commonality of phenotype, we postulated that the dramatic change seen with multiple reverting agents could indicate the existence of specific molecular ‘nodes’ that control architectural reorganization in response to this great variety of microenvironmental factors.

Our analyses revealed that the extensive common differences in the gene expression profile of cells with an organized and disorganized phenotype in mammary epithelial cells are associated with at least one transcriptional regulator, NFkB. This makes NFkB one of the critical nodes through which the microenvironmental signaling pathways pass/integrate. When NFkB is suppressed, the architecture is maintained and tissues remain functional. Should the microenvironmental signaling be disrupted, NFkB becomes activated, and both tissue polarity and normal functions become imbalanced. In our studies blocking NFkB with a chemical inhibitor or shRNA was sufficient for the reestablishment of basal polarity in malignant cells; conversely in all conditions where malignant breast cancer cells were ‘reverted’, NFkB levels were decreased.

NFkB has been shown to be involved in the development and progression of breast cancer: It is frequently highly activated in mammary tumors, in particular within disorganized ER-negative tumors where it has been reported to promote cancer progression by enhancing cancer cell survival and proliferation [27, 28]. To this list we now add an additional important function of NFkB as an important player in loss of tissue polarity and organization. It was shown previously that modulation of many different oncogenic pathways including EGFR, PI3K and integrin signaling leads to changes in NFkB activity [29-35]. Also, activation of the NFkB pathway is required for induction and maintenance of EMT in mammary tumors [36]. In our 3D model, down-modulation of all these pathways leads to a reorganized phenotype, and our analysis suggests that NFkB is a major molecular linker between these pathways and tissue architecture: Of the 24 genes that are upregulated in disorganized cell clusters and that have potential NFkB binding sites in their promotor region (Supplemental Table 3, marked in yellow), four genes were associated with actin cytsoskeletal organization, cell junction and cell adhesion in the GO database (*ACTN1, EPB41L2, GNE, PANX1*).

This has strong implications for NFkB in cancer prevention and as target for therapeutic efforts: If keeping NFkB balanced is the key to healthy mammary tissues, signaling gone awry in malignant cells can be compensated through reducing other stimuli activating NFkB, including reductions in stress and inflammation. As long as NFkB is high, integration of the aberrant signaling cannot be restored to normal levels. As a consequence, compensatory signaling pathways can be easily activated in cells that have become imbalanced. We suggest that inhibition of NFkB in breast cells could be one crucial change that would allow these cells to reorganize and thereby to re-integrate their signaling. The additional details of how exactly the microenvironmental signals reach NFkB and what are the other arms by which the switch occurs require further investigation.

## MATERIALS AND METHODS

### Cell culture

HMT-3522 S1 and T4-2 cells were maintained on tissue culture plastic as previously described [7]. BT549 cells were propagated in DMEM/F12 (Sigma) with 10% fetal bovine serum (Invitrogen). Three dimensional laminin-rich extracellular matrix (3D lrECM) on-top cultures [37] were prepared by trypsinization of cells from tissue culture plastic, seeding of single cells on top of a thin gel of Engelbreth-Holm-Swarm (EHS) tumor extract (Matrigel: BD Biosciences; Cultrex BME: Trevigen), and addition of medium containing 5% EHS. S1 cells were seeded at a density of 3.1×10^4^ cells per cm^2^; T4 and BT549 cell lines were seeded at 2.1 ×10^4^ cells per cm^2^. S1 and T4-2 were maintained in their propagation medium with media change every 2 days. BT549 cells were maintained in H14 medium [7] with 1% fetal bovine serum.

### Microarray hybridization and analysis

S1, T4-2 and reverted T4 cells were isolated from 3D cultures with PBS/EDTA as previously described [37]. Purified total cellular RNA was extracted using RNeasy Mini Kit with on-column DNase digestion (Qiagen). RNA was quantified by measuring optical density at A260 and quality was verified by agarose gel electrophoresis. Affymetrix microarray analysis was performed using the Affymetrix HG-U133A High Throughput Array (HTA) GeneChip system, generating two sets of data from two separate array platforms (GPL3921 and GPL4685, GEO database GSE50444). Preprocessing, normalization and filtering was performed using R Bioconductor [38].MAS5 normalization was performed separately on the two sets of expression data. ProbeIDs were filtered out if the average log2 expression value was lower than 6.5. For genes with more than one probeID, we only considered the one with the highest variance. The two sets of expression values were row-centered (mean and variance) separately and then merged to one gene expression matrix. Unsupervised hierarchical clustering analysis was performed with Cluster (uncentered correlation, average linkage), and results were visualized with TreeView (http://www.eisenlab.org/eisen/?page_id=42). Gene set enrichment analysis was performed with GSEA v2.07 (http://www.broadinstitute.org/gsea/index.jsp, [21]). Network analysis was performed with Cytoscape 2.7.0. Transcription factor binding site analysis was performed using TransFind (−300 to 100 bp, top 500 genes, [22]). Potential targets of NFkB transcriptional regulation were retrieved by repeated transcription factor binding site analysis with subsets of the disorganization signature using Transfind. DAVID Gene Ontology was used to determine overrepresented GO and KEGG terms [19].

### Virus production and transduction

For p65 shRNA production, a double-stranded DNA oligonucleotide was generated from the following sequences: sense,5'-GATCCGGACATATGAGACCTTCAACTTCCTGTCAGATATATCTCTCCTTCCACACTTTTTG-3'; antisense,5'-AATTCAAAAAGGACATATGAGACCTTCAATCTGACAGGAAGTATATCTCTCCTTCCACACG-3' (target sequence underlined; BamH1/EcoR1 cohesive ends italicized). Both oligonucleotides were annealed and ligated into BamH1/EcoR1 site of pGreen puro lentival vector (System Biosciences). Lentivirus production and transduction of target cells were conducted following guideline by System Biosciences. Briefly, lentivirus vector and packaging plasmid mix (System Biosciences) were transfected into 293FT cells (Invitrogen) using Lipofectamine® 2000. After 48 hours, medium was harvested, filtered and used to infect target T4-2 cells with the addition of polybrene (10 μg/ml). After 24 hours medium was replaced. At 72 hours post-infection puromycin (0.5 μg/ml) was added for selection and maintained throughout the culturing period. shRNA vector against RelB were from Open Biosystems. Retrovirus production and infection protocol from the company were followed.

### Immunofluorescence and image acquisition

Cells in lrECM gel were smeared on slides, dried briefly, and fixed with 4% paraformaldehyde and permeabilized with 0.5 % Triton X-100. Stained samples were imaged using a Spot RT camera attached to a Zeiss upright epifluorescence microscope or a Stanford Photonics XR/Mega-10 ICCD camera attached to a Solamere Technology Group (Salt Lake City, UT) spinning disk confocal system comprised of a Zeiss Axiovert 200M inverted microscope. Pictures were taken using a 63×oil immersion objective with QED InVivo imaging software at room temperature. The digital images were pseudocolored, overlayed and merged using ImageJ 1.38 or Adobe Photoshop 7.0.

### RT-PCR and real-time PCR

Total RNA was extracted from cells using RNeasy Mini Kit (Qiagen). cDNA was synthesized using Superscript first strand synthesis kit (Invitrogen) from 0.5-1.0 μg RNA samples. Quantitative real-time PCR analysis was performed with the Lightcycler System using the Lightcycler FastStart DNA Master SYBR Green I kit (Roche). The following Lightcycler PCR amplification protocol was used: 95°C for 10 min (initial denaturation), and 45 amplification cycles (95°C for 5 s, 60°C for 10 s, 72°C for 5 s). Amplification was followed by melting curve analysis to verify the presence of a single PCR product [39].

### Luciferase assays and western blot analysis

Three copies of NF-κB response element were linked to TK minimal promoter and cloned into a reporter vector pGL3 (Promega). T4-2 cells were co-transfected with pGL-NF-κB and pNeo plasmid (1:10). Stably transfected cells were isolated by G418 selection and cultured in 3D in the presence of Tyrphostin. Following treatment, equal amounts of cell lysates were assayed for luciferase activity.

S1 and T4-2 cells were cultured in 3D for 10 days. Total and nuclear protein was extracted from S1, T4-2, and reverted T4 cells or nuclei. Western blot experiments were performed as previously described [40]. After sonication, insoluble material was removed by centrifugation at 15,000 g for 10 min. Proteins (20 μg) from each sample were subjected to SDS gel electrophoresis and then transferred to nitrocellulose membrane (Schleicher & Schuell). The membrane was subsequently incubated in blocking buffer containing primary antibody and horseradish peroxidase-conjugated secondary antibodies. The membrane was then subjected to enhanced chemiluminescence (ECL) using the SuperSignal chemiluminescent substrate (Pierce, Rockford, IL).

## Supplementary Figures and Tables




