# Supplementary material for: NFkB disrupts tissue polarity in 3D by preventing integration of microenvironmental signals
Source: Oncotarget. 2013 Oct 14;4(11):2010–20. doi: 10.18632/oncotarget.1451 (PMC3875766; doi:10.18632/oncotarget.1451)
Supplement: Supplementary file 1 [file oncotarget-04-2010-s001.pdf]

NFkB disrupts tissue polarity in 3D by preventing integration of microenvironmental signals - Becker-Weimann et al

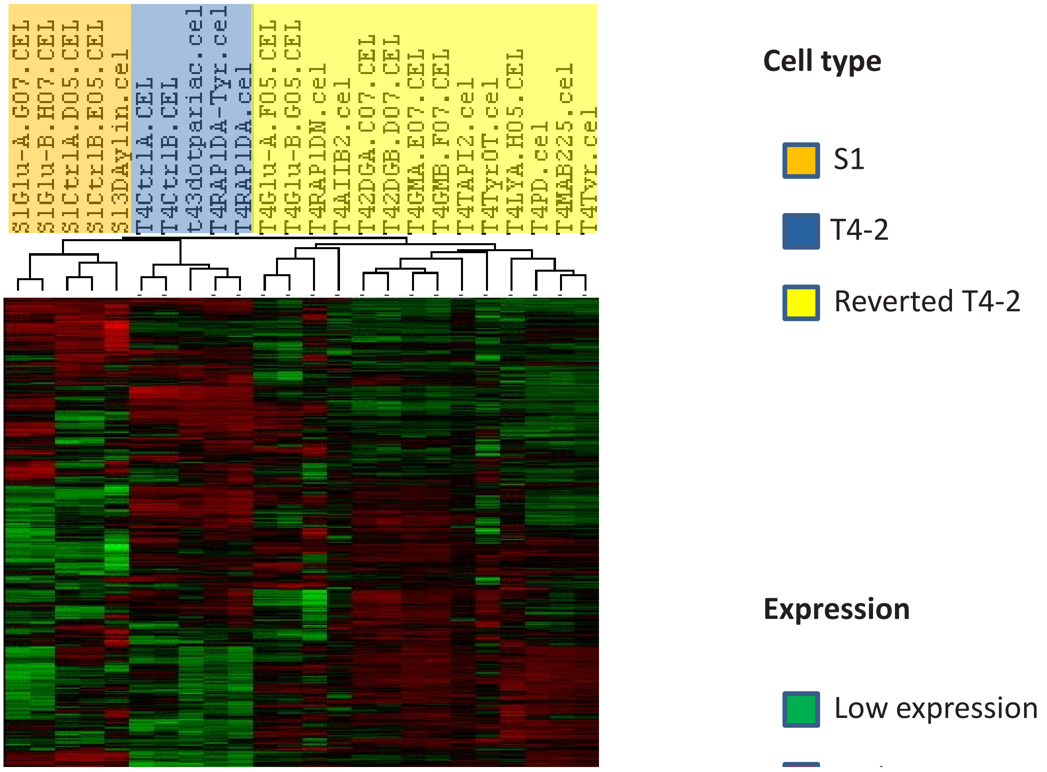

Figure S1: Unsupervised hierarchical clustering of the mRNA expression matrix consisting of 6560 genes and 24 samples from S1, T4-2 and reverted T4-2 cells.

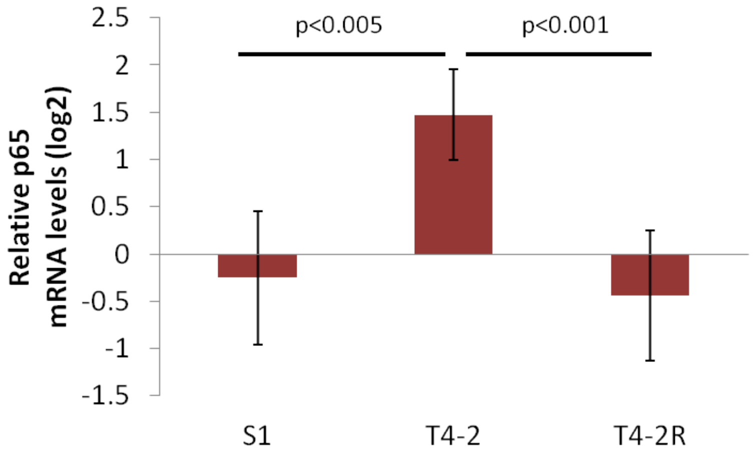

Figure S2: p65 mRNA levels in S1, T4-2 and reverted T4 cells. p65 mRNA expression is assessed by Affymetrix microarray and measured as log2(probe intensities).

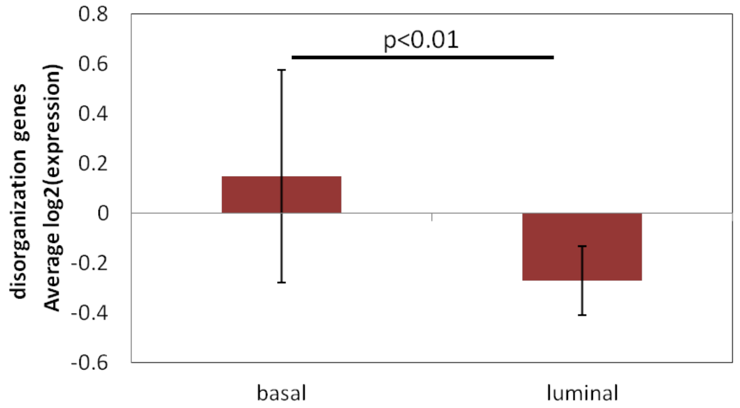

Figure S3: Box plot of average RelB mRNA levels in basal and luminal cell lines. RelB mRNA expression is assessed by Affymetrix microarray and measured as log2(probe intensities).

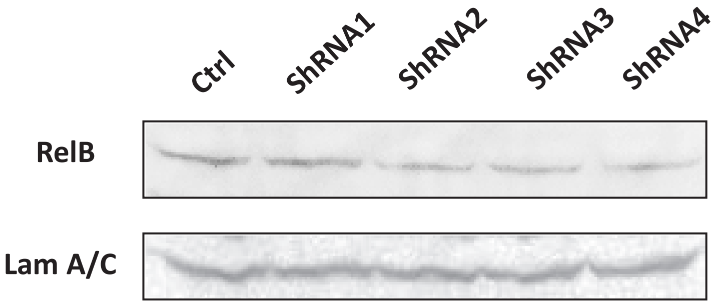

Figure S4: Immunoblot analysis of RelB expression in RelB shRNA-expressing BT549 cells. shRNA 4 has relatively strong knockdown effects, and was therefore chosen for the following experiments.
